# Pilomatrix carcinoma of the right postauricular region: A case report and literature review

**DOI:** 10.1016/j.ijscr.2019.10.087

**Published:** 2019-11-06

**Authors:** Shabiah Martin, Jana DeJesus, Ann Jacob, Teah Qvavadze, Claudio Guerrieri, Rachel Hudacko, Thaddeus Boucree

**Affiliations:** Newark Beth Israel Medical Center, 201 Lyons Ave, Newark, NJ 07112, USA

**Keywords:** Pilomatrix carcinoma, Malignant pilomatrixoma, Wide local excision, Local recurrence, Case report

## Abstract

**Introduction:**

Pilomatrix carcinoma is a rare aggressive tumor with a high rate of local recurrence after surgical excision. Diagnosis is made by histopathology and when discovered, wide local excision has been shown to have the best results.

**Presentation of case:**

We report a case of a 74-year-old male incidentally found to have a large right postauricular mass and regional lymphadenopathy. The mass was biopsied and proven to be a malignant pilomatrixoma. Wide local excision and level II and III neck dissection with reconstruction using a right supraclavicular flap was performed.

**Discussion:**

Pilomatrix carcinoma is a lesion first described in 1880 by Malherbe and Chenantais. It is unknown if these tumors arise de novo or arise through malignant transformation of a benign pilomatrixoma. There are similarities between the benign lesion and its malignant counterpart in terms of activating mutations in signaling pathways. A well-defined gold standard for surgical management has not been established, but currently wide local excision with safe margins is recommended along with regional lymph node dissection when metastasis is suspected. Currently, no chemotherapy regimen has been shown to be effective in local control or in preventing metastatic spread.

**Conclusion:**

Pilomatrix carcinoma, given its aggressive nature, has a high propensity for recurrence after excision. It is important to perform wide local excision to avoid an incomplete resection and higher recurrence rates. Further studies will be needed to create a more defined standard of treatment and to evaluate the role of adjuvant chemotherapy and radiation therapy.

## Highlights

• Pilomatrix carcinoma is a very rare locally aggressive tumor.
• A well-defined gold standard for surgical management has not been established.
• Currently wide local excision with safe margins is recommended.
• Regional lymph node dissection is performed when metastasis is suspected.

## 1 Introduction

Pilomatrix carcinoma is a rare, locally aggressive tumor arising from the hair follicle matrix. It commonly presents as an asymptomatic non-tender, firm, mobile nodule, most often in the head and neck region [1,2]. These lesions are typically asymmetric with a bluish discoloration and may have ulceration of the overlying skin [[3], [4], [5]]. The benign form has a higher incidence in children aged 8 years to 13 years and has a female predominance, occurring at a 3:2 ratio [6]. Malignant pilomatrixomas typically occur in the 5^th^ to 7^th^ decades of life and are seen more frequently in males with a 3:1 ratio [5,7]. The diagnosis of pilomatrical tumors is made by histopathology. They are characterized by nests of basaloid cells with trichilemmal-type keratinization and “shadow” or “ghost” cells in the center, with or without calcification and foreign body giant cell reaction to keratin debris [4,6]. The benign tumor is well-circumscribed with a capsule of connective tissue and is usually located in the deep dermis and subcutaneous fat. The malignant counterpart, or pilomatrix carcinoma, is distinguished from the benign form by the presence of hyperchromatic vesicular atypical basaloid cells with squamous metaplasia, increased mitotic activity, necrosis, infiltrative borders, lymphovascular invasion, and invasion into surrounding structures [8].

Unlike benign pilomatrixomas which have low recurrence rates after surgical excision, malignant pilomatrixomas are locally aggressive and have a high rate of recurrence after surgical excision [6]. Due to its rare occurrence, a well-defined gold standard of management has not been established, and the roles of further treatment post-excision are yet to be defined. We present a case of a pilomatrix carcinoma of the postauricular area in a patient who presented to an inner city teaching hospital for unrelated symptoms. This case report has been reported in line with the SCARE criteria [9].

## 2 Presentation of case

**Fig. 1:**
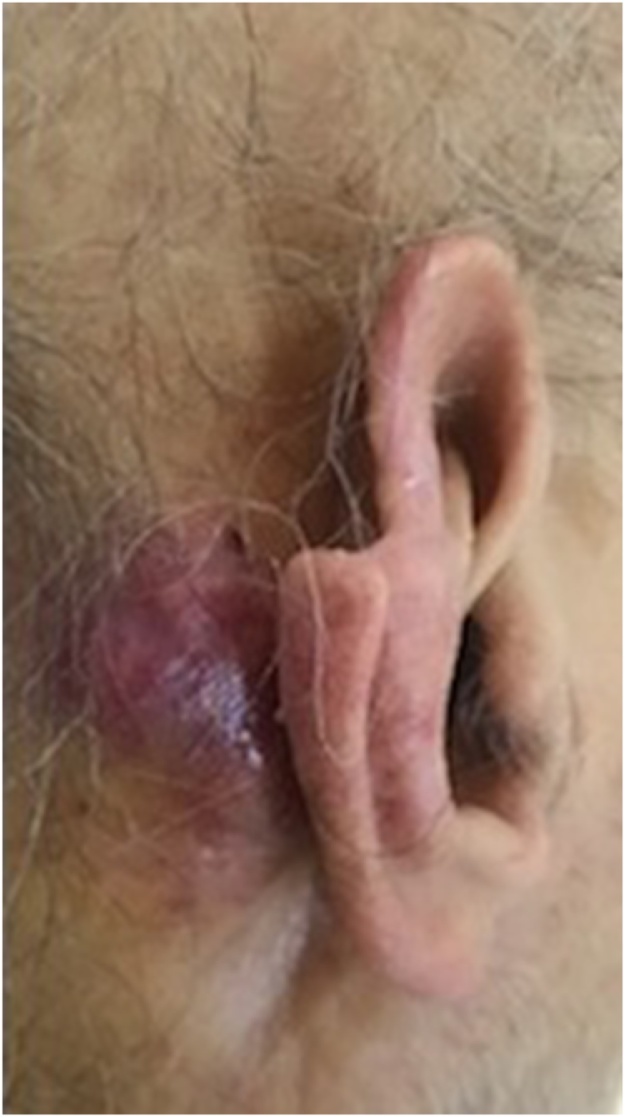
Malignant pilomatrixoma as a firm ovoid mass in postauricular area. The helical defect on the ear is from a previously resected squamous cell carcinoma.

**Fig. 2:**
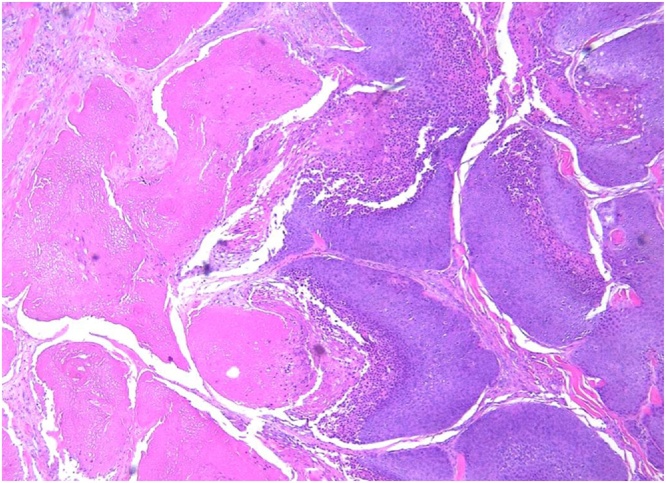
Incisional biopsy showing areas composed of nests of basaloid epithelial cells (right) with abrupt keratinization and anucleated ghost cells (left). H&E stain, 4×. These are typical findings in a pilomatrixoma.

**Fig. 3:**
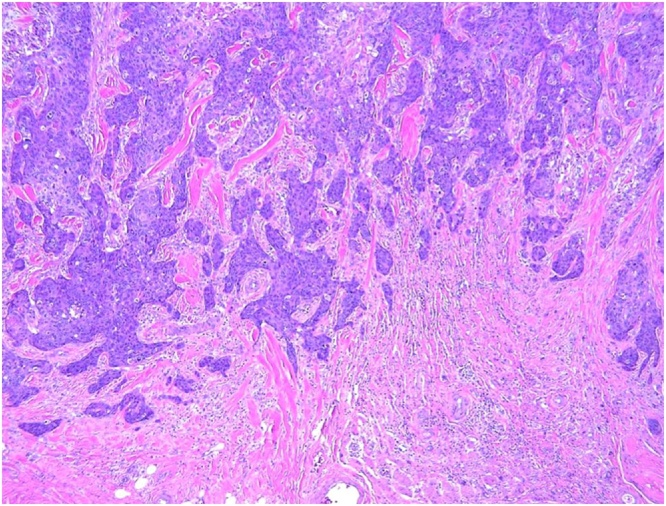
At the periphery of the tumor, the nests of epithelial cells showed a more squamoid appearance with irregular infiltrative borders. H&E stain, 10×.

**Fig. 4:**
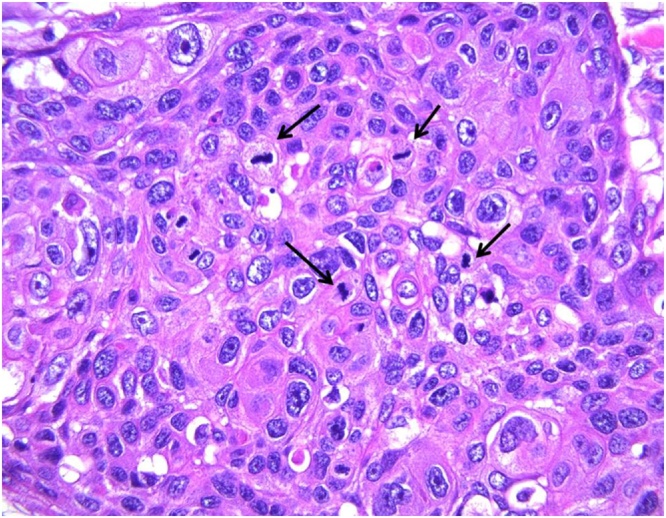
On high power, the squamoid cells showed marked cytologic atypia with enlarged irregular nuclei, prominent nucleoli, and increased mitotic figures (arrows). H&E stain, 40×.

**Fig. 5:**
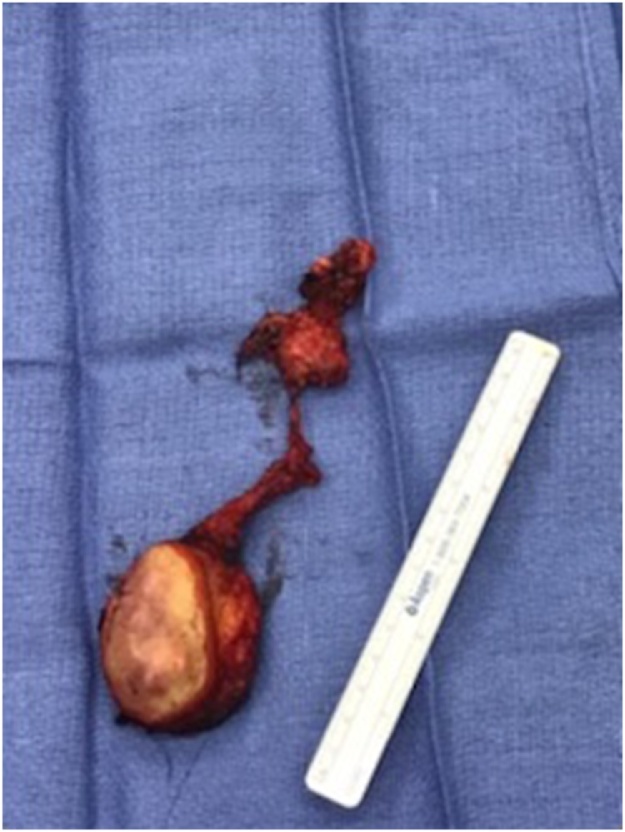
Excised postauricular mass in continuity with fibrofatty tissue from posterior triangle. The mass was tan and firm and measured 4.3 × 3 × 2.2 cm.

A 74-year-old male with a past medical history significant for congestive heart failure, orthotopic heart transplant eight years prior, a previously resected squamous cell carcinoma of the right ear, diabetes type II, chronic kidney disease, and severe neutropenia presented for workup of a syncopal episode. He was noted to have a large elliptical mass measuring approximately 4 cm located in the right postauricular area with associated regional lymphadenopathy (Fig. 1). The patient underwent imaging studies which were negative for metastatic disease. An ultrasound-guided core biopsy of the lesion was performed, showing a few small clusters of atypical epithelial cells consistent with a carcinoma. The tissue was insufficient for further diagnostic evaluation, and the patient underwent an incisional biopsy. The incisional biopsy demonstrated a dermal pilomatrixoma (Fig. 2) with cytologic atypia, squamoid differentiation, infiltrative borders, up to 50 mitoses per 10 high-power fields, and atypical mitotic figures (Fig. 3, Fig. 4). These findings were consistent with a malignant pilomatrixoma. After his syncopal workup was complete, surgical excision was planned. Wide local excision of the mass with level II and III neck dissection was performed. The postauricular mass was resected in continuity with fibrofatty tissue from the posterior triangle (Fig. 5). Eight cervical lymph nodes were obtained. Reconstruction of the postauricular area was performed using a right supraclavicular flap.

Pathological examination of the resected specimen demonstrated the presence of basaloid cells with squamous metaplasia, cytologic atypia, significant mitotic activity, and necrosis. The lesion was present in the dermis and subcutaneous tissue and lacked an epidermal component. These findings were consistent with a malignant pilomatrixoma. The tumor was present at the deep margin of resection and was within 0.1 mm of the lateral margin of resection. Eight lymph nodes were negative for metastatic carcinoma. The patient was scheduled for postoperative radiation therapy but expired due to liver failure secondary to congestive heart failure.

## 3 Discussion

Pilomatrix carcinoma is a rare malignant and locally aggressive hair follicle tumor with a high propensity for recurrence after excision [1,7,10]. It is largely unknown if these lesions arise de novo or through malignant transformation of a pre-existing benign pilomatrixoma [10,11]. Pilomatrixoma was first described in 1880 by Malherbe and Chenantais as a “calcifying epithelioma” that was thought to be derived from the sebaceous gland [12]. The earliest known description of the malignant counterpart was by Gromiko in 1927 [13]. A literature review by Christopher, et al. examined 125 recorded cases of pilomatrix carcinoma. Of those, 10 arose in a background of histologically confirmed benign pilomatrixoma [14]. Similar activating mutations of the Wnt signaling pathway have been found in both benign and malignant pilomatrixomas suggesting the possibility of malignant transformation [15,16]. A study performed by Lazar et al. in 2005 found a possible association of these tumors with mutations of the beta-catenin gene (CTNNB1). Both benign and malignant lesions showed increased expression of beta-catenin, suggesting malignancy arises from a precursor benign lesion [11]. Mutations in the CTNNB1 gene, which encodes beta-catenin and is a downstream effector in the Wnt pathway acting as a signal for cell differentiation and proliferation, leads to the development of tumors of hair matrix differentiation [5,10,[15], [16], [17]]. Beta-catenin gene mutations have been reported in the vast majority of pilomatrixomas, and beta-catenin is essential in E-cadherin function [18]. Invasive characteristics of this lesion develop when the expression or structure of E-cadherin fails [18]. Given this finding, it is possible that beta-catenin mutations result in the development of pilomatrixomas, and over time, additional mutations lead to malignant transformation [16].

When comparing benign and malignant pilomatrixomas, both can be well-circumscribed with nucleated basaloid cells at the periphery transitioning to anucleated shadow or ghost cells in the center. Both can have calcification and foreign body giant cell reaction signifying a granulomatous response to the keratinized debris. One characteristic difference is that pilomatrix carcinoma shows a proliferation of pleomorphic hyperchromatic basaloid cells with areas of squamous metaplasia and exhibits numerous mitoses and prominent nucleoli. Pilomatrix carcinomas also have an infiltrative growth pattern and can have necrosis and lymphovascular invasion [8,14].

Since pilomatrix carcinoma is a rare entity, a well-defined gold standard for surgical management has not been established. Most authors recommend wide local excision with safe margins of 0.5 cm–1 cm as opposed to simple excision, as an incomplete resection results in high recurrence rates [7,15]. In patients who undergo simple excision, the recurrence rate is noted to be up to 50–60% [16]. Herman et al combined published literature with their series of cases and reported a 23% recurrence rate in tumors that were widely excised, in contrast to an 83% recurrence rate in tumors that were simply excised [15]. In their series, along with all previously reported cases, metastases occurred in 13% of cases, with regional lymph nodes, lungs, bone, other visceral organs, and the brain being the most common sites [15]. In most cases, metastatic disease was noted at or after the time of local recurrence, and in a few cases, metastatic disease was discovered at the time of diagnosis or without the presence of clinical recurrence [15]. Lymph node metastases should be treated with regional lymph node dissection in combination with wide local excision of the primary lesion [6].

The role of adjuvant chemotherapy and radiotherapy in treatment has yet to be fully defined and remains unclear. Neither treatment modality has shown a significant difference in modifying the disease course. Adjuvant radiation therapy is recommended for locoregional, metastatic, and recurrent disease, or if there are questions concerning clear margins [1,8,15,19,20]. However, no chemotherapy regimen has been shown to be effective in local control or in preventing metastatic spread [8,19,20].

## 4 Conclusion

Pilomatrix carcinoma is a rare malignant and locally aggressive hair follicle tumor with a high propensity for recurrence after excision. A well-defined gold standard for surgical management has not yet been established. In patients who undergo simple excision, the recurrence rate is noted to be high. Most authors recommend wide local excision over simple excision, as an incomplete resection results in a higher rate of recurrence. Lymph node metastases should be treated with regional lymph node dissection. The role of adjuvant chemotherapy and radiotherapy has yet to be fully defined and remains unclear. In the case presented, the patient underwent wide local excision, and all lymph nodes were negative for metastasis. Unfortunately, the patient expired due to unrelated reasons, thus adjuvant treatment and monitoring for recurrence could not be performed.

## Sources of funding

No funding received.

## Ethical approval

This case report was discussed with the IRB department at Newark Beth Israel Medical Center. No IRB review or exemption is required.

## Consent

Written informed consent was obtained from the patient for publication of this case report and accompanying images. A copy of the written consent is available for review by the Editor-in-Chief of this journal on request.

## Author’s contribution

Shabiah Martin – Corresponding author; First author; Review and editing.

Jana DeJesus – Co-Author.

Ann Jacob – Co-Author.

Teah Qvavadze – Resources.

Thaddeus Boucree – Resources.

Claudio Guerrieri – Review and editing; Resources.

Rachel Hudacko – Co-Author; Review and editing; Resources.

## Registration of research studies

N/A.

## Guarantor

Shabiah Martin.

## Provenance and peer review

Editorially reviewed, not externally peer-reviewed.

## Declaration of Competing Interest

No conflicts of interest to disclose.
